# Keratinocyte cytoskeletal roles in cell sheet engineering

**DOI:** 10.1186/1472-6750-13-17

**Published:** 2013-02-26

**Authors:** Qi Wei, Daniel Reidler, Min Ye Shen, Hayden Huang

**Affiliations:** 1 Department of Biomedical Engineering, Columbia University, 351 Engineering Terrace, 500 W 120th Street, MC 8904, New York, NY 10027, USA

**Keywords:** Cell sheet, Cytoskeleton, Adhesion, Contraction

## Abstract

**Background:**

There is an increasing need to understand cell-cell interactions for cell and tissue engineering purposes, such as optimizing cell sheet constructs, as well as for examining adhesion defect diseases. For cell-sheet engineering, one major obstacle to sheet function is that cell sheets in suspension are fragile and, over time, will contract. While the role of the cytoskeleton in maintaining the structure and adhesion of cells cultured on a rigid substrate is well-characterized, a systematic examination of the role played by different components of the cytoskeleton in regulating cell sheet contraction and cohesion in the absence of a substrate has been lacking.

**Results:**

In this study, keratinocytes were cultured until confluent and cell sheets were generated using dispase to remove the influence of the substrate. The effects of disrupting actin, microtubules or intermediate filaments on cell-cell interactions were assessed by measuring cell sheet cohesion and contraction. Keratin intermediate filament disruption caused comparable effects on cell sheet cohesion and contraction, when compared to actin or microtubule disruption. Interfering with actomyosin contraction demonstrated that interfering with cell contraction can also diminish cell cohesion.

**Conclusions:**

All components of the cytoskeleton are involved in maintaining cell sheet cohesion and contraction, although not to the same extent. These findings demonstrate that substrate-free cell sheet biomechanical properties are dependent on the integrity of the cytoskeleton network.

## Background

The development of cell-sheet tissue engineering, where cells are plated and allowed to form confluent layers which are then dissociated from the plate to form intact, functional sheets, has generated a need for a systematic characterization of cell-cell interactions to better condition constructs for in vivo use [1-3]. Such cell sheets have been generated for a wide variety of tissues, such as skin, heart, corneal and renal components [4-6]. Cell sheets generated for tissue engineering purposes are fragile and are typically handled by using external supports, such as chitin membranes [7]. Methods for improving the strength and other mechanical properties of such sheets is essential for further development of these constructs. However, to be effective, such methods must rely on information regarding the mechanism by which sheet properties are regulated. For example, of interest would be mechanisms by which cell sheet contraction is limited by targeting select aspects of the cell cytoskeleton. To uncover such mechanisms, there needs to be a systematic examination of the role of the cytoskeleton in regulating cell sheet properties. Further, there is a significant amount of recent interest in the relationship between the cytoskeleton and cell-cell interactions to model physiology or disease processes [8-10].

The cellular cytoskeleton primarily consists of three main components in mammalian cells – actin, microtubules and intermediate filaments. For cells that remained attached to a substrate, the contribution of the cytoskeleton to cell-substrate adhesion, spreading, and signaling have been extensively studied [11-21]. Actin is a well-examined cytoskeletal component, since actin links to the focal adhesion complex and disruption of actin is linked to reduced traction forces and altered mechanotransductive signaling [16,22-26]. Microtubules have a role in supporting the actin framework and destabilizing focal adhesions [27,28], but play more prominent roles in cell division and intracellular transport. Intermediate filaments are much less frequently examined, but are thought to be involved in tissue strength [29-32]. Much less is known about the roles of these components in determining the properties of suspended cell sheets, however.

While many previous studies in cell–sheet engineering use thermoresponsive polymers, the use of dispase to generate intact cell sheets can also be used to measure generate cell sheets for examination [33-36]. The relative impact of each component of the cytoskeleton on cell sheet cohesion and cell sheet contraction is not currently well-established. Additionally, passive and active contraction might be involved in sheet contraction, but the relative role of each is still poorly understood. Contraction may influence the sheet’s ability to provide sufficient coverage in the tissues being repaired. However, inhibition of contraction by interfering with acto-myosin contraction may also influence sheet strength. Thus, there is a need for systematic characterization for the role of the cytoskeleton in regulating cell sheet strength and contraction.

To address this need, we disrupt cytoskeletal components in cell sheets to assess the effects of such disruption on cell sheet cohesion and contraction. We show that all three main components of the cytoskeleton are distributed differently in cell sheets, contribute to cell sheet cohesion strength and contraction. Additionally, our previous work suggests that cell sheet contraction is a mechanism for parts of the cell cytoskeleton to reinforce cell-cell junctions [36]. Inhibition of such contraction may weaken this reinforcement, leading to more fragile cell sheets. Keratinocytes were chosen for this study in part due to existing interest in keratinocyte cell sheet cohesion for dermal tissue engineering and in part because they exhibit strong cell-cell interactions, including desmosomes, which provide a solid foundation for including intermediate filaments in the consideration of cell sheet properties. Together, these findings demonstrate that the biomechanical properties of substrate-free cell sheet may be dependent on the integrity of the cytoskeleton network. This study of substrate-free cell sheet contraction and response to cytoskeleton disruption represents a quantitative investigation of the link between cell sheet cytoskeletal biology and mechanics.

## Results

### Dispase lifting and cytoskeletal disruption variably alter cytoskeletal distribution

Since some key mechanical properties of cells are dependent on the cellular cytoskeleton, we first assessed changes in cytoskeletal distribution in cell sheets lifted from a plating substrate, as well as from disruptions of specific cytoskeletal components.

**Figure 1:**
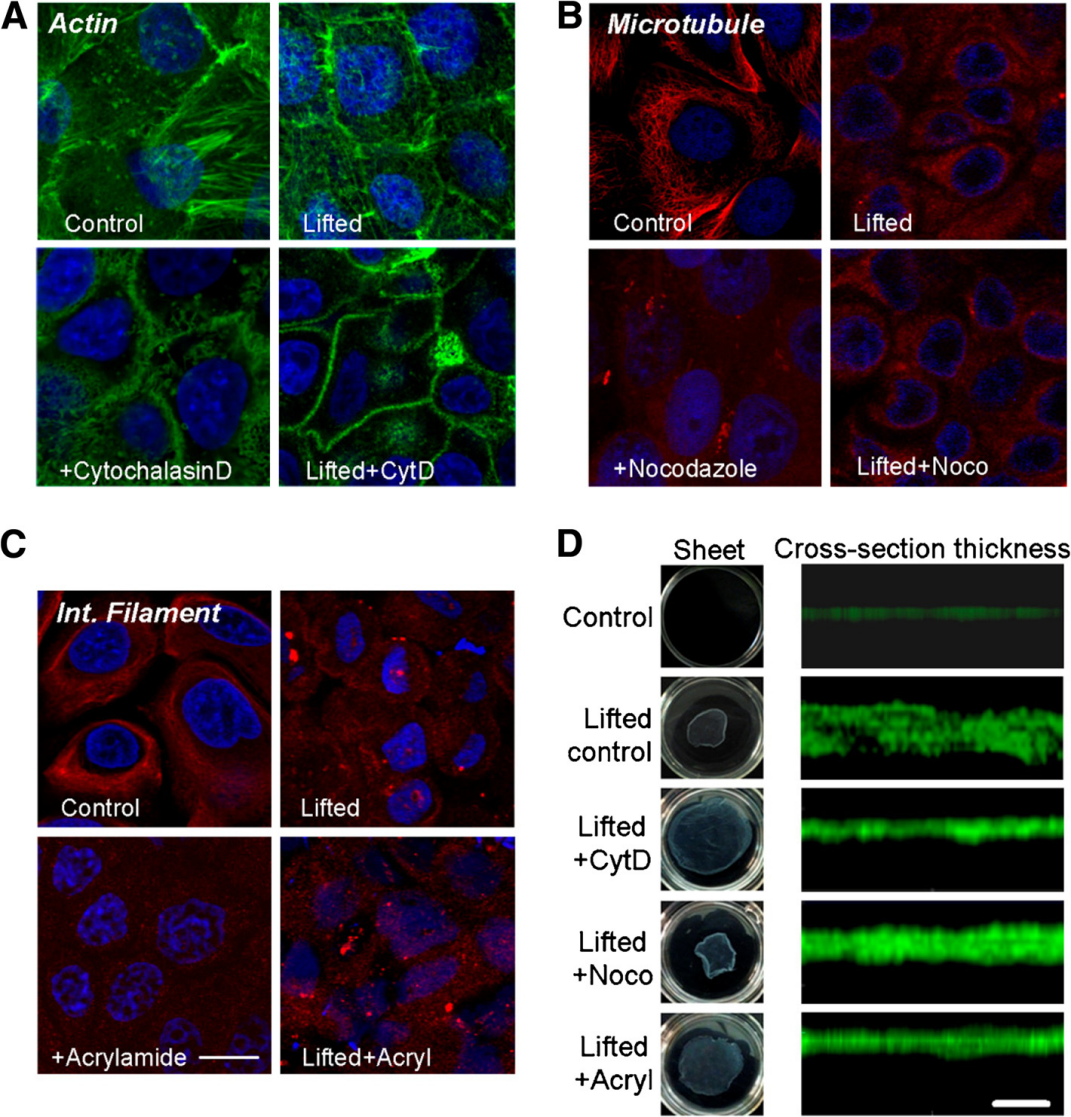
**Dispase dissociation and cytoskeletal disruption alter cytoskeletal distribution.** Cytoskeletal labeling of (**A**) actin, (**B**) microtubules and (**C**) keratin intermediate filaments in keratinocytes under conditions of control, lifted, toxin treatment, and lifting + toxin treatment. The keratin stain using Monoclonal Anti-Cytokeratin, pan antibody was very dense and thus individual filaments are not visible easily. Scale bar is 10 μm. (**D**) Whole sheet images in 35 mm dishes and cross-sectional thickness images under conditions of control, dispase-lifted, toxin treatment, and lifting + toxin treatment. Cells were stained with CellTracker Green. Scale bar is 50 μm.

Phalloidin staining of untreated, attached cells shows actin localized predominantly at cell-cell junctions with some stress fibers in the cytoplasmic region of the cells (Figure 1A, top left image). Dispase-lifted monolayers exhibit contraction, resulting in cells displaying smaller horizontal cross-sections. Actin remained localized to the cell-cell junctions (Figure 1A, top right image). Both prior to (Figure 1A, bottom left image) and post dispase-dissociation (Figure 1A, bottom right image), treatment of the cells with actin-disrupting cytochalasin-D altered, but did not eliminate, the junctional localization of actin, with only diffuse staining visible in the cytoplasmic region of the cells.

Cells labeled for microtubules exhibit dense, convoluted fibers with minimal junctional localization (Figure 1B, top left image). Dispase-lifted monolayers display a loss of fibers with diffuse staining (Figure 1B, top right image). After treatment with microtubule disrupting nocodazole, the cells display diffuse staining, even while adhered to a substrate (Figure 1B, bottom left image). Dispase-lifting the nocodazole-treated cells did not lead to any significant further changes, compared to nocodazole-treated adhering cells, other than cell contraction (Figure 1B, bottom right image). In dispase-lifted conditions, there is a notable lack of signal at cell-cell junctions, not due to cell-cell separation, suggesting that microtubules may be excluded from those regions.

The intermediate filament keratin staining (using Monoclonal Anti-Cytokeratin, pan antibody) exhibits somewhat uniform staining with few fibers throughout the cytoplasm, with little apparent junctional localization (Figure 1C, top left image). Dispase lifting leads to diffuse cytoplasmic staining with some focal deposits (Figure 1C, top right image). Intermediate filament-disrupting acrylamide treatment results in loss of visible fibers (Figure 1C, bottom left image), while lifted and acrylamide-treated cell sheets exhibit similar loss of fibers, but with some contraction (Figure 1C, bottom right image).

After dispase-lifting, cell sheets exhibits contraction immediately on separation from the substrate, and cytoskeletal disruption affects the degree of sheet contraction (Figure 1D). Sheet thickness measurements also exhibits different thickness with cytoskeletal disruption, with sheet thickness for plated control (5.9 ± 1.5 μm), dispase-lifted control (25.5 ± 3.7 μm), dispase-lifted + CytD treatment (14.2 ± 2.1 μm), dispase-lifted + nocodazole treatment (20.9 ± 2.6 μm) and dispase-lifted + acryl treatment (17.0 ± 1.7 μm).

### Cytoskeletal disruption weakens cell sheet cohesion

**Figure 2:**
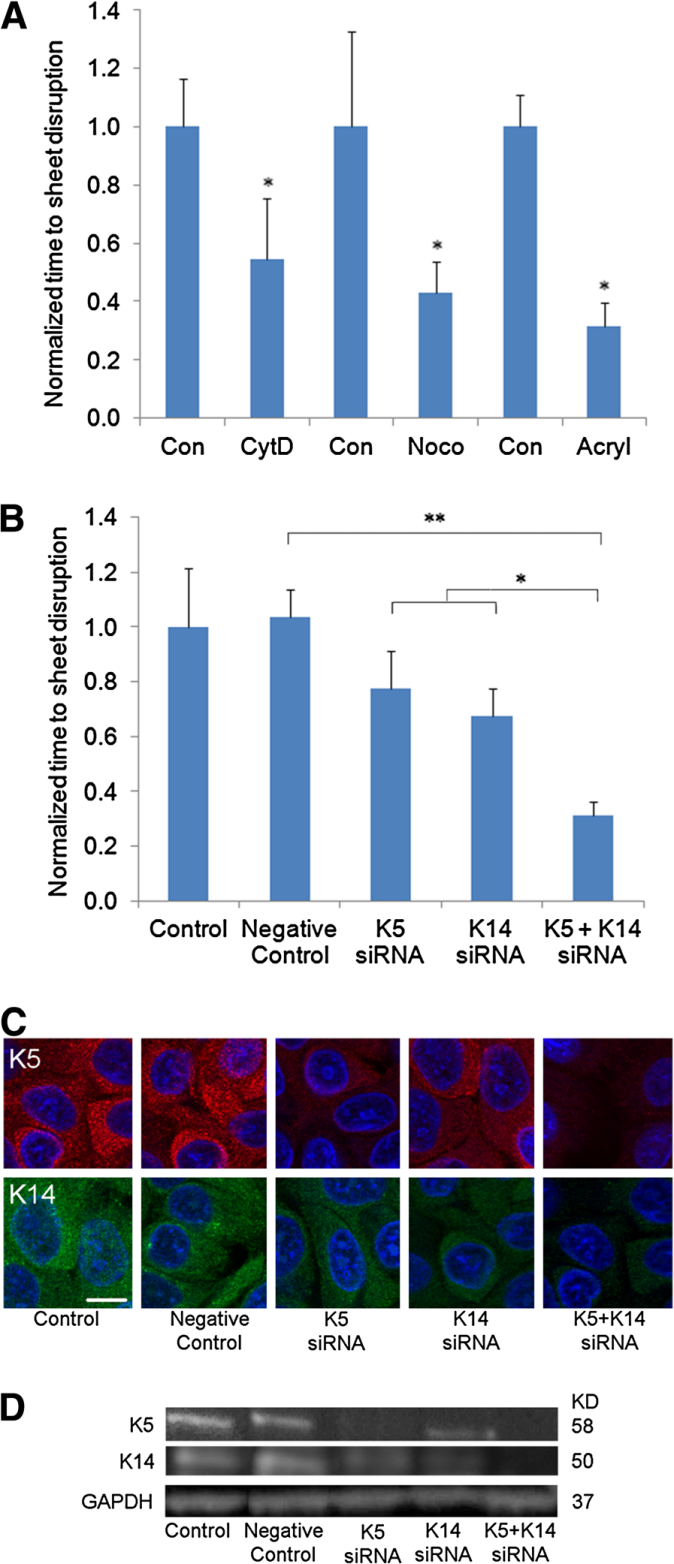
**(A) Shear test measuring cell sheet cohesion shows significant diminishment in cohesion strength with disruption of each component of the cytoskeleton using toxins, with acrylamide treatment exhibiting the greatest decrease in cell sheet cohesion.** *p<0.05 compared to the matched untreated control. Unnormalized times in seconds: con/CytD=140/73; con/noco=93/40; con/acryl=148/45. (**B**) Shear test shows double siRNA knockdown of keratin 5 and keratin 14 weakens cell sheet cohesion. *p<0.05 and **p<0.01. Unnormalized times in seconds: control=145; negative control=150; K5=113; K14=98; K5+K14=45. (**C**) Immuno-staining and (**D**) western blot analysis of siRNA transfection on adhered cells demonstrates reduction of K5 or K14 expression levels. K5/K14 knockdown together significantly reduced both K5 and K14 expression levels. Scale bar is 10 μm. GAPDH was used as a loading control.

We next assessed the cohesion strength of cell sheets to determine how individual cytoskeletal components may regulate cell sheet cohesion. Shear tests were performed on cell sheets with actin, microtubule, or intermediate filament disruption and compared to matched untreated controls, with increased disruption time indicating increased cell sheet cohesion (Figure 2A; unnormalized times in seconds: con/CytD=140/73; con/noco=93/40; con/acryl=148/45). Disruption of any of the cytoskeletal components weakens cell sheet cohesion. While acrylamide-treated sheet appears to be weaker than sheets treated with either cytochalasin-D or nocodazole, this effect is not statistically significant (p>0.05). Although acrylamide is known to disrupt intermediate filaments in some cells [37], the specificity of acrylamide on intermediate filaments is not without controversy. Because the keratin pair K5/K14 has been reported as reliable markers for the study of keratin organization in keratinocytes [38,39], we next examined the role of K5/K14 in modulating cell sheet cohesion.

RNA interference was used to knockdown K5 and/or K14 (Figure 2B). Shear tests were performed on siRNA treated cell sheets compared to matched untreated controls, with increased disruption time indicating increased cell sheet cohesion (Figure 2B; unnormalized times in seconds: control=145; negative control=150; K5=113; K14=98; K5+K14=45). No difference was observed between control and the sample treated with negative control siRNA (p>0.05). Knockdown of K5 or K14 alone exhibited a non-statistically significant reduction in disruption time compared to control or the negative control siRNA treated sample (p>0.05). Knockdown of K5 and 14 together exhibited significantly reduced disruption time compared to either control (p<0.01) or knockdown of K5 or K14 alone (p<0.05). Notably, the knockdown of K5 and K14 together had a similar effect as acrylamide.

Immunofluorescence staining (using K5/8 and K14 monoclonal antibodies) on adhered cells four days after siRNA transfection showed diminished keratin expression, and combined knockdown of K5/14 together exhibited a larger reduction of keratin expression. No visible difference was observed between control and the sample treated with the negative control siRNA (Figure 2C). To confirm the effects of siRNA knockdown of keratins, western blot analysis of siRNA transfection was performed, and showed reduction of K5 or K14 expression levels (Figure 2D). Similar results were obtained using a second set of K5 and K14 siRNA (Additional file 1: Figure S1).

### Dispase lifting results in cytoskeletal-dependent cell sheet contraction

**Figure 3:**
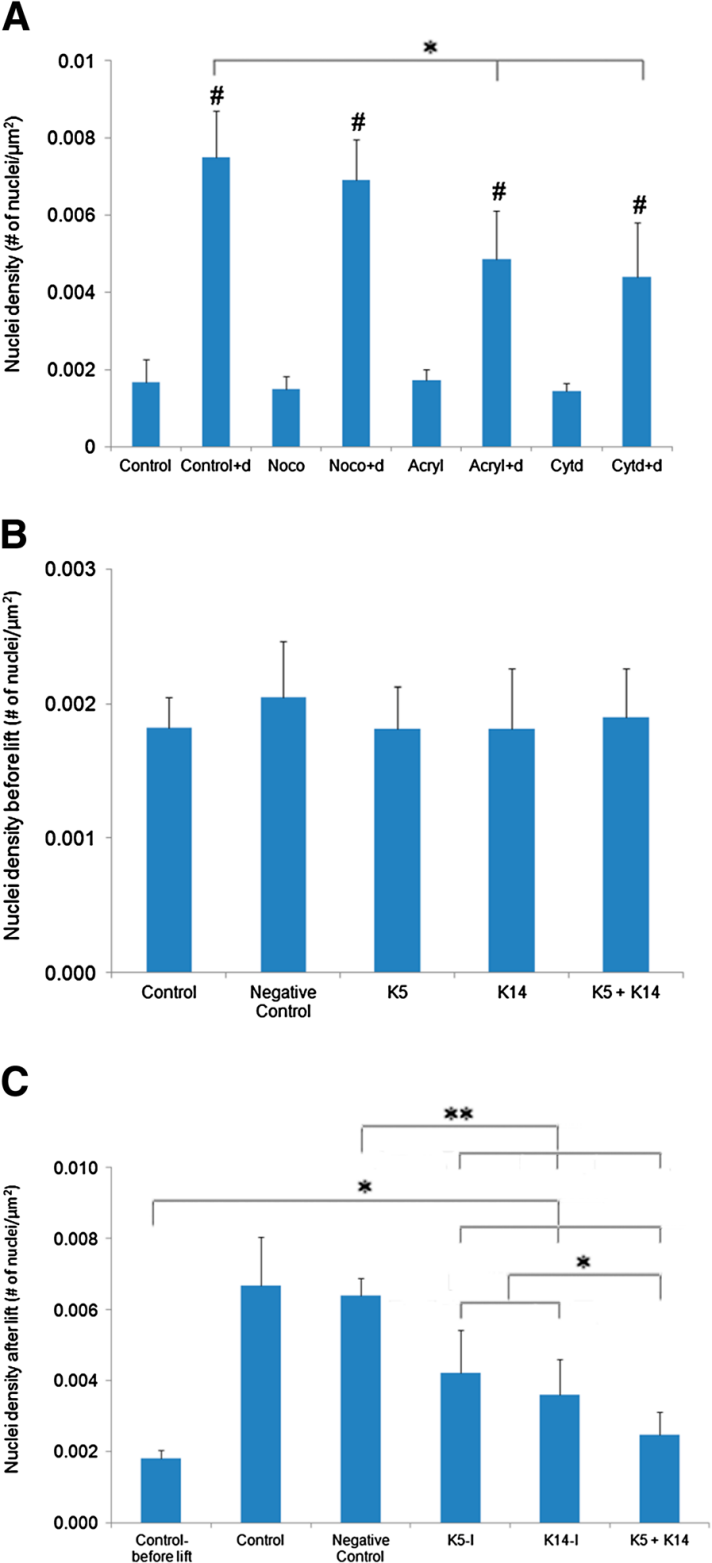
**(A) Cell sheets contract after treatment and dispase lifting.** For each treatment, the dispase-lifted cell sheet exhibited a significant increase in nuclei density compared to the unlifted cells, indicating cell sheet contraction (#p<0.05 for the baseline case versus the matched baseline+d case). The notation “+ d” indicates the condition after dispase treatment. (**B**) After siRNA transfection to suppress either K5, K14 or both, without dispase lifting, no significant difference in nuclear density was observed among all treated samples. (**C**) After dispase lifting the siRNA transfected samples, all sheets contracted, but K5 and K14 siRNA treated contracted significantly less than the sample treated with negative control siRNA (*p<0.05, **p<0.01).

All cell sheets exhibited significant contraction when lifted off the culture dish (Figure 3A, p<0.05 for all cases compared to unlifted controls), using nuclear density as quantification. Control cells showed a large amount of contraction post dispase lift. Nocodazole-treated cells exhibited comparable contraction as controls (p>0.05, control + dispase versus nocodazole + dispase). Both cytochalasin-D and acrylamide treated cells exhibited reduced contraction (p<0.05 for control + dispase versus cytochalasin D + dispase or acrylamide + dispase).

Contraction assays performed using K5/K14 siRNA transfection showed no significant difference in nuclear density when cells remain attached to the cell culture dish (Figure 3B). Immediately after dispase lifting, all samples exhibited significant increases in nuclei density and thus significant contraction, relative to the pre-lifted control (Figure 3C, p<0.05). Controls and the sample treated with scrambled negative control siRNA showed the greatest amount of contraction (p>0.05 control versus negative control after dispase lift). Both K5 and K14 siRNA treated samples exhibited reduced but significant contraction (p<0.01 for K5, K14 versus control before lift). Combined treatment with K5 and K14 siRNA together exhibited further reduced contraction (p<0.01 for K5+K14 versus control-before lift; p<0.05 for K5+K14 versus each of K5 or K14). Further, K5 and/or K14 siRNA treated samples contracted significantly less than the sample treated with scrambled negative control siRNA (p<0.01 for K5, K14, K5+K14 versus negative control). Similar results were obtained using a second set of K5 and K14 siRNA (Additional file 2: Figure S2).

Individual keratinocyte diameters were measured on trypsinized cells (i.e., single cells in suspension). The average cell diameter was found to be 18±2 μm. If we assume the cells are roughly spherical, then the volume of the cell can be calculated from that diameter as 3,054±339 μm^3^. The average thickness of a spread (control) cell can be calculated by multiplying the nuclei density (Figure 3A) by the cell volume to get 5.1±1.9 μm for the control cell and 22.9±4.5 μm for a dispase-lifted cell. With cytoskeletal disruption, the dispase-lifted cell sheet thicknesses are 21.1±4 μm with nocodazole, 13.4±4.6 μm with cytochalasin D, and 14.8±4.2 μm with acrylamide. These values correlate quite well with the cross-sectional thickness measured using cell sheet images (Figure 1D). The force of cell contraction in a dispase lifted control sheet compresses the cell so that the cell height is greater than the cell’s lateral dimension, indicating the cells have columnar morphology when dissociated from the cell plating surface. This effect is reduced in cell sheets with actin and keratin suppression.

### Disruption of actomyosin contraction affects both cell contraction and cell sheet cohesion

**Figure 4:**
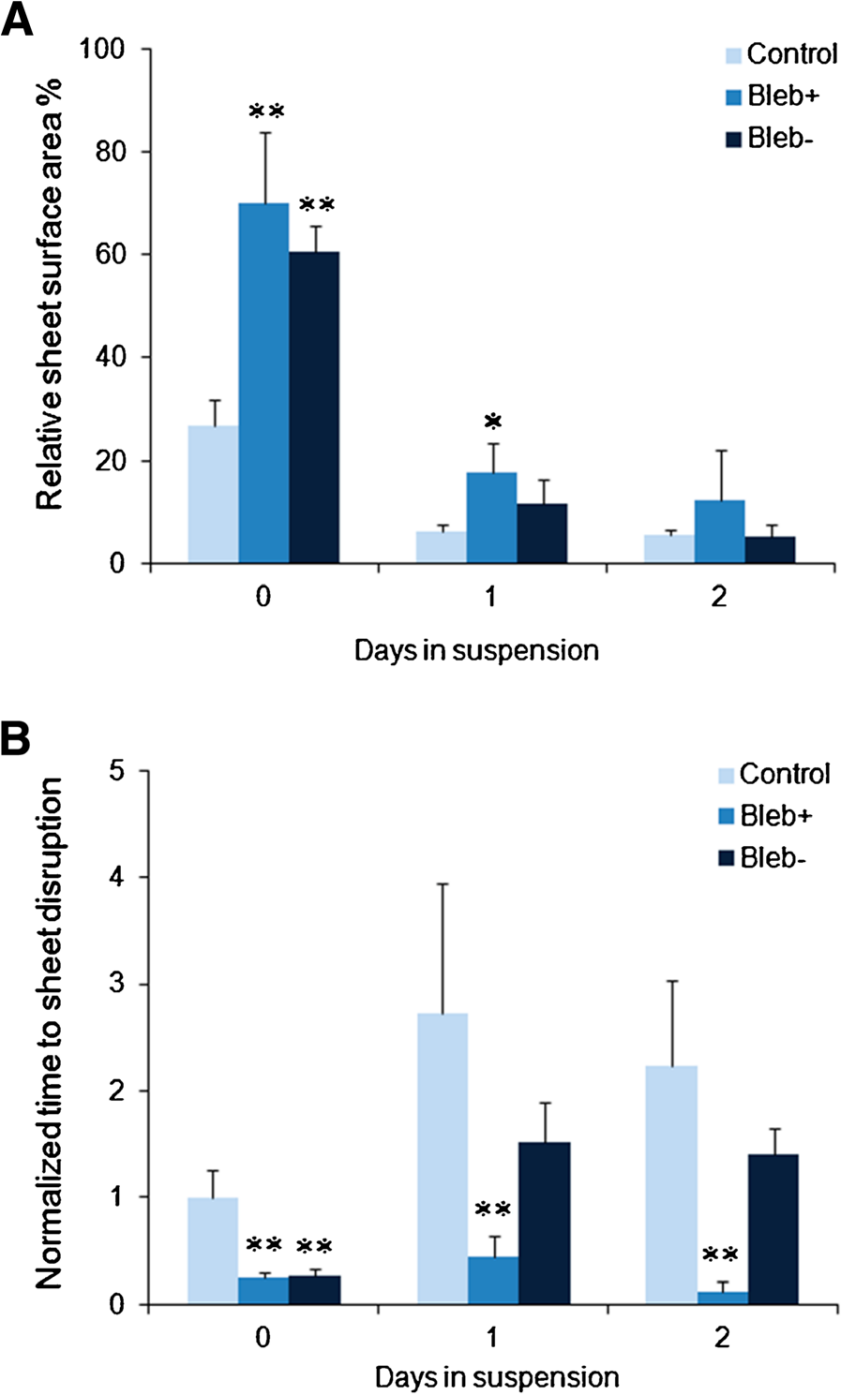
**(A) Cell sheet contraction under control, continued blebbistatin treatment (bleb+) or blebbistatin treatment for one day prior to dispase lifting, followed by reversion to normal media (bleb-) demonstrates that while blebbistatin does limit the degree of cell sheet contraction upon initial dispase lifting, the effects rapidly wear off and have no long-term effect on the degree of contraction.** (**B**) Cell sheet cohesion is markedly reduced on blebbistatin treatment, but rapidly recovers on removal of blebbistatin. *p<0.05, **p<0.01 compared to the matched untreated control. Unnormalized times in seconds: control 0/1/2=93/253/208; bleb+ 0/1/2=23/41/11; bleb- 0/1/2=24/140/131.

To determine the role of actomyosin contraction on cell sheet contraction and cohesion, cells were treated with blebbistatin for one day prior to dispase lifting, followed by either continued blebbistatin treatment or replacement back to normal media (bleb+ and bleb-, respectively) and compared against control (no blebbistatin treatment, Figure 4A). Pretreatment with blebbistatin ensures that actomyosin interactions are inhibited upon dispase lift and permits the assessment of actomyosin’s role in the initial contraction of cell sheets. Immediately upon dispase-lifting (day 0), control cell sheets shrunk to about 20-25% of their original area, consistent with a four-to-five-fold increase in nuclei density reported in Figure 3. This contraction was markedly decreased with blebbistatin treatment, which resulted in contraction to 60-70% of their original area (p<0.05 for both blebbistatin treatments at day 0). After one day of incubation in suspension, control cell sheets contracted even further to 6% of the original area. The cell sheets that were maintained in blebbistatin-dosed media (bleb+) contracted to 18% of the original area, but were still significantly greater than the time-matched control (p<0.05). In contrast, in the cell sheets incubated with blebbistatin, but reverted to normal media (bleb-) for one day, exhibited similar contraction as control (p>0.05). By two days post dispase lifting, all of the cell sheets (control, bleb+ and bleb-) exhibited similar areas, indicating that the effects of continued blebbistatin dosing cannot permanently halt cell sheet contraction.

We next assessed whether blebbistatin affects cell sheet cohesion using shear tests (Figure 4B; Unnormalized times in seconds in (B): control 0/1/2=93/253/208; bleb+ 0/1/2=23/41/11; bleb- 0/1/2=24/140/131). Immediately upon dispase lifting (day 0), blebbistatin-treated cell sheets were considerably more fragile compared to control cells (p<0.05). After one day of treatment, bleb- cell sheets exhibited cohesion strength comparable to that of control cells, which themselves exhibited increased cohesion (compared to controls at day 0). However, the bleb+ sheet was considerably more fragile compared to control (p<0.05). After a second day of incubation, bleb- cell sheets continued to match control levels of cohesion, while bleb+ cell sheets essentially broke apart right away with nominal shears, indicating significantly reduced cohesion compared to control (p<0.05). These results indicate that disrupting actomyosin contraction interferes with both contraction and cell sheet cohesion, even without directly disrupting the cytoskeletal fibers.

## Discussion

Understanding cell-cell interactions is important for generating strategies for conditioning cell sheets or other constructs for tissue engineering purposes, or at least to define some limitations of cell-sheet engineering. Further, characterizing cell-cell interactions are crucial for modeling the balance between cell-cell and cell-substrate interactions. Thus, results from the characterization of how different cytoskeletal components contribute to cell sheet cohesion and contraction have broad application.

To determine the contribution of different parts of the cytoskeleton to cell sheet mechanics, we assessed the changes to the cytoskeleton from dispase lifting as well as from pharmaceutical or siRNA-based disruption of each of the major cytoskeletal components. Consistent with the idea that the intermediate filaments contribute to tissue strength, we show that keratins are at least as important as actin in establishing cell sheet cohesion, even though actin clearly localizes to cell-cell junctions, whereas microtubules and intermediate filaments appear less prominent at these junctions. Disruption of microtubules also diminishes cell sheet strength, suggesting that all components of the cytoskeleton likely play significant roles in establishing cell sheet cohesion. While increased cell sheet fragility may also be due to cell rupture (similar to what is observed in blistering diseases), the role of the cytoskeleton remains vital to keeping the cell sheet whole.

Dispase lifting resulted in cell sheet contraction, with disruption of actin or intermediate filaments, but not microtubules, significantly reducing the amount of cell sheet contraction. Since actin is a major component generating traction forces against a substrate and is involved in cell-cell linkages via cadherins, it is not unexpected that actin disruption also diminishes cell-cell contractile force. Interestingly, microtubules do not appear to have a major role in regulating cell sheet contraction, while they influence cell sheet strength. The contribution of intermediate filaments has the same, or greater, magnitude as actin, suggesting that there is a contractile mechanism that depends on intermediate filaments. Additionally, the (untreated) cell-cell contractile force is of magnitude sufficient to create a columnar morphology of the cells, where cell-cell contact area is vastly increased due to the increased thickness. Such a phenomenon may be a result of compensation from loss of cell-substrate adhesion, but we show that it can be reversed by certain types of cytoskeletal disruption.

Blebbistatin treatment significantly reduced cell sheet contraction, and this effect was transient if blebbistatin was removed. Further, blebbistatin treatment also significantly reduced cell-sheet cohesion. Removal of blebbistatin restored contraction and cell sheet cohesion within a day of removal. However, maintaining cell sheets in blebbistatin did not eliminate the effects of contraction over time. Further, cell sheet cohesion remains reduced, even as the sheet contracts. We hypothesize that continued cell sheet contraction is via an intermediate filament mechanism which is unaffected by blebbistatin, but the cell sheet cohesion component that relies on actin is somehow disrupted by blebbistatin’s action, resulting in reduced cohesion as the cells try to contract with reduced cell-cell junctional reinforcement. Testing this hypothesis is difficult, however, due to the increased fragility of cell sheets with intermediate filament disruption in addition to blebbistatin treatment. Elucidation of this, or some other, contraction mechanism would likely yield further insight into the role of the cytoskeleton in regulating cell-cell interactions.

Both passive and active processes are likely involved in sheet contractions. One possible model is that initial contraction on dispase lifting is generated primarily by passive processes, perhaps resulting from prestressed actin and intermediate filaments (but not microtubules). Continued contraction of the cell sheet over the next 48 hours results from active processes (although passive viscoelastic processes may still contribute). Our results indicate that inhibiting actomyosin contraction does not eliminate this extended contraction period, suggesting that other mechanisms, such as molecular motors, may contribute during active contraction. Interestingly, the degree of contraction resulting from passive processes is about the same as for active processes (both reduce area by roughly a factor of 4). Further clarification of passive versus active contraction may lead to alternative approaches to modify the size and strength of these sheets.

In this study we show that all components of the cytoskeleton are important for regulating the properties of the cell sheet, but that in particular the intermediate filaments, which are generally not well-studied, play a significant role in maintaining cell sheet cohesion and potentially in regulating cell sheet contraction. Thus, optimization of cell sheet engineering may benefit from manipulation of all three components of the cytoskeleton, such as via mechanical conditioning. Combined with our previous work on keratinocytes sheets [36], we can propose the following for tissue-engineering applications. If cells sheets are initially too fragile for use, extended incubation in suspension will yield improved cohesion at the cost of usable area. The amount of contraction is to roughly a quarter of the original sheet area if used immediately. Alternatively, if cell sheet cohesion is extremely strong, then pretreatment with either actomyosin-, actin- or keratin-disrupting compounds will yield increased area at the cost of increased fragility. Microtubule disruption yields increased fragility without a corresponding preservation in cell sheet size and thus is likely not useful to creating larger, stronger sheets.

## Conclusions

The primary conclusions of this study are (1) substrate-free cell sheet biomechanical properties are strongly dependent on the integrity of the cytoskeleton network and interactions between neighboring cells, (2) cytoskeletal distributions change in substrate-free cell sheets, (3) actin and intermediate filaments, and microtubules to a lesser extent, regulate cell sheet cohesion and contractility of cell sheets, (4) actomyosin interactions also regulate those properties, and (5) both active and passive contractions are involved in cell sheet contraction. These findings suggest that cytoskeleton and tightly associated intercellular junctions may be crucial for unlocking the potentials for cell sheet engineering, which has emerged as a promising approach to reconstructing various types of laminar tissues, such as skin, myocardium, cornea, and vascular systems, etc. without using any biodegradable scaffolds.

## Methods

### Reagents and materials

Unless otherwise stated, media and reagents were from Invitrogen (Carlsbad, CA). Hoechst 33342 was used to perform live-cell nuclear staining at a dilution of 1:4000 in cell media for 45–60 minutes at 37°C. Monoclonal anti-β-tubulin (Sigma, St. Louis, MO) was used at a dilution of 1:400. Monoclonal Anti-Cytokeratin, pan antibody (PCK-26, Sigma) and Monoclonal Anti-Keratin 5/8 antibody (C50, sc-8021, Santa Cruz Biotechnology), Monoclonal Anti-Keratin 14 antibody (LL001, sc-53253, Santa Cruz Biotechnology) were used at a dilution of 1:1000. Alexa Fluor 594 goat anti-mouse IgG was used at a dilution of 1:1000. Alexa fluor 488 phalloidin was used at a dilution of 1:40. Cytochalasin D (cytD, Sigma) was used to disrupt actin filaments at a concentration of 3 μM for one hour. Nocodazole (noco, Sigma) was used to disrupt the microtubules at a concentration of 10 μM for one hour. Acrylamide (acryl, Sigma) was used to disrupt keratin filaments at a concentration of 10 mM for four hours. Blebbistatin (Sigma) was used at 100 μM for up to 72 hours. Immunofluorescence staining was performed using the mouse monoclonal anti-plakoglobin antibody (γ-Catenin, Sigma) as the primary and Alexa Fluor 594 goat anti-mouse IgG as the secondary antibody at a dilution of 1:1000.

### Cell culture

Immortalized human keratinocytes (N/TERT-1) were maintained as described elsewhere [36,40]. Briefly, cells were expanded and propagated in keratinocyte serum-free media (abbreviated ker-sfm), supplemented with rEGF (0.2 ng/ml) and BPE (25mg/ml), CaCl_2_ (Sigma, St.Louis, MO, 0.4mM), and penicillin/streptomycin. To grow cells to high confluency, cells were switched to a medium consisting of a 1:1 mixture of ker-sfm and a medium DF-K, the latter consisting of a 1:1 mixture of DMEM and Ham’s F-12, supplemented with rEGF (0.2 ng/ml) and BPE (25mg/ml), L-glutamine (1.5 mM) and penicillin/streptomycin.

Dispase was applied to confluent cells at a concentration of 2.4 units/mL in Hanks Buffered Saline Solution (HBSS) and incubated at 37°C until the cell sheet lifted.

Fixation of either adhered or dispase-lifted cell sheets did not significantly alter the spacing of the cells. Addition of DMSO as a vehicle control did not result in significant differences compared to non-DMSO controls in the contraction and shearing assays.

### siRNA knockdown of keratin

Two Silencer Pre-designed siRNA for keratin 5 (first siRNA sequence5’-3’: GCAUGUCUCUGACACCUCAtt; second siRNA sequence 5’-3’: GGAGAGUAGUCUAGACCAAtt), two Silencer Pre-designed siRNA for keratin 14 (first siRNA sequence 5’-3’: GCCGAGGAAUGGUUCUUCAtt; second siRNA sequence 5’-3’: GGACAUGGAUGUGCACGAUtt), and a negative control siRNA (Invitrogen) were used to assess the effects of keratin 5 and keratin 14 inhibition on cell sheet contraction and cell sheet cohesion. Cells were transfected with siRNA using Lipofectamine 2000 (Invitrogen), according to the manufacturer’s instructions. Transfection was performed using 100pmol siRNA oligomer or 100 pmol DNA plasmid and 6 μl Lipofectamine 2000 for each sample in a 35 mm dish. Other size formats were scaled up or down accordingly. Cells were lysed and analyzed for keratin 5 and keratin 14 by immunoblotting four days after transfection. Results presented in figures were from the first silencer sequence, but was repeated with the second as well, which gave similar results.

### Fluorescence microscopy

Cells were fixed in 4% paraformaldehyde (Sigma) and then permeabilized with 0.1% triton-X-100 (Sigma) or 100% methanol at −20°C. Cells were incubated in the primary antibody or phalloidin, CellTracker Green (CTGreen) and/or Hoechst label for an hour and then, for samples tagged with a primary antibody, a secondary antibody incubation for another hour. Microscopy images were acquired with an Olympus IX-81 inverted fluorescence microscope using 10x NA 0.13 and 40x NA 0.60 objective lenses, and an Orca CCD (Hamamatsu, Bridgewater, NJ, model C10600) camera using MetaMorph Software. Additional images were acquired using an Olympus FV10 Confocal microscope, an Olympus OPLFLN 40X O NA 1.3 objective lens, and Olympus FV10-ABW Software. Imaging was performed on the central sheet regions, where the sheets were mostly flat. Images were processed using ImageJ (version 1.43u for Windows; National Institutes of Health) and scaled down in Photoshop (Adobe) to prepare the final figures. Images were colored and adjusted for brightness and contrast.

Fixed cell sheets (post dispase lifting) labeled with CellTracker (Invitrogen) were imaged with confocal microscopy. Cell sheet thickness was determined by measuring random vertical lines across the sheets. At least six measurements per sheet across two sheets were used for each condition.

### Cell sheet contraction assay

A confluent monolayer of keratinocytes was labeled with Hoechst and imaged. The number of nuclei in fixed subregions of the images were counted and normalized by area to obtain nuclei density, in nuclei per square microns. This process was repeated for a confluent monolayer that was dispase lifted from the dishes to assess the contraction of the cell sheet. The use of subregions was necessitated by focal issues from cell sheets not being completely flat.

Whole-sheet contraction was measured without nuclei labeling during blebbistatin experiments, and Microsoft Paint was used to quantify cell sheet diameters and subsequently, sheet areas. Sheet areas were then used to compare the effect of blebbistatin on sheet contraction. The results were normalized to each time-matched control. Nuclei readouts were not used since Hoechst is toxic to cells long-term and the blebbistatin experiments lasted up to three days.

### Shear test to measure cell-cell adhesion

A dispase-based lifting assay was performed to test cell sheet strength, and thus cell-cell adhesive strength, adapted from well-established similar protocols [33-36]. Cells grown to full confluence in 35 mm culture dishes were treated with dispase until the monolayer lifted from the dish as an intact cell sheet. Cell sheets were carefully transferred to 50 ml tubes containing 10 ml of media, then vortexed at a fixed setting at 10 second intervals. The earliest time for which the cell sheet was completely disrupted, as assessed visually, was recorded for each sheet. Shear test times were normalized to control values due to differences in cell growth rates, leading to variability in control shear time from experiment to experiment.

### Immunoblotting

Total protein concentration was determined by Bradford assay (Sigma). Soluble fractions containing equal amounts of total protein were separated using SDS polyacrylamide gel electrophoresis and transferred onto PVDF membranes (Perkin Elmer, Waltham MA). Immunoblotting was performed using mouse anti-human antibodies diluted in TBS with 0.1% v/v Tween-20 (Sigma) at the following dilutions: anti-keratin 1:1,000, anti-GAPDH 1:1,000, and horseradish peroxidase conjugated goat anti-mouse secondary antibody 1:1,000. Blots were developed with ECL reagents (Perkin Elmer) and imaged using a FUJI imaging unit (Fujifilm, Stamford, CT).

### Statistics

ANOVA was performed on the cell sheet contraction data. The shear test results were analyzed using t-tests. In all cases, a p-value of 0.05 or lower was considered significant. Where applicable, data are presented as mean ± standard deviation. At least 3 sheets were used for each contraction and cohesion assay.

## Competing interest

The authors declare no competing interests.

## Author’s contributions

QW carried out staining, cell experiments and analysis. DR carried out cell experiments. MS carried out staining. HH conceived of the study and drafted the manuscript. All authors helped determine the direction of the article, edited the manuscript and approved its submission.

## Supplementary Material

Additional file 1: Figure S1 K5 and K14 siRNA expression and cohesion assay results using the second siRNA. (A) Shear test measuring cell sheet cohesion shows significant reduction in cohesion strength with double keratin knockdown. *p<0.05 and **p<0.01. Unnormalized times in seconds: control=145; negative control=150 (same controls as for Figure 2A); K5-II=120; K14-II=96; K5-II + K14-II=50. Immunostaining images of (B) K5 knockdown and (C) K14 knockdown. Scale bar is 10 μm. Western Blot analysis of (D) K5 knockdown and (E) K14 knockdown on adhered cells demonstrates reduction of K5 or K14 expression levels. GAPDH was used as a loading control. Click here for file

Additional file 2: Figure S2 K5 and K14 siRNA contraction assay results using the second siRNA. (A) After siRNA transfection to suppress K5, K14, or both, without dispase lifting, no significant difference in nuclear density was observed among all treated samples. (B) After dispase lifting the siRNA transfected samples, all sheets contracted, but K5 and K14 siRNA treated contracted significantly less than the sample treated with negative control siRNA. *p<0.05, **p<0.01. Click here for file
