# Supplementary figures and images for: Keratinocyte cytoskeletal roles in cell sheet engineering
Source: BMC Biotechnol. 2013 Feb 26;13:17. doi: 10.1186/1472-6750-13-17 (PMC3599259; doi:10.1186/1472-6750-13-17)

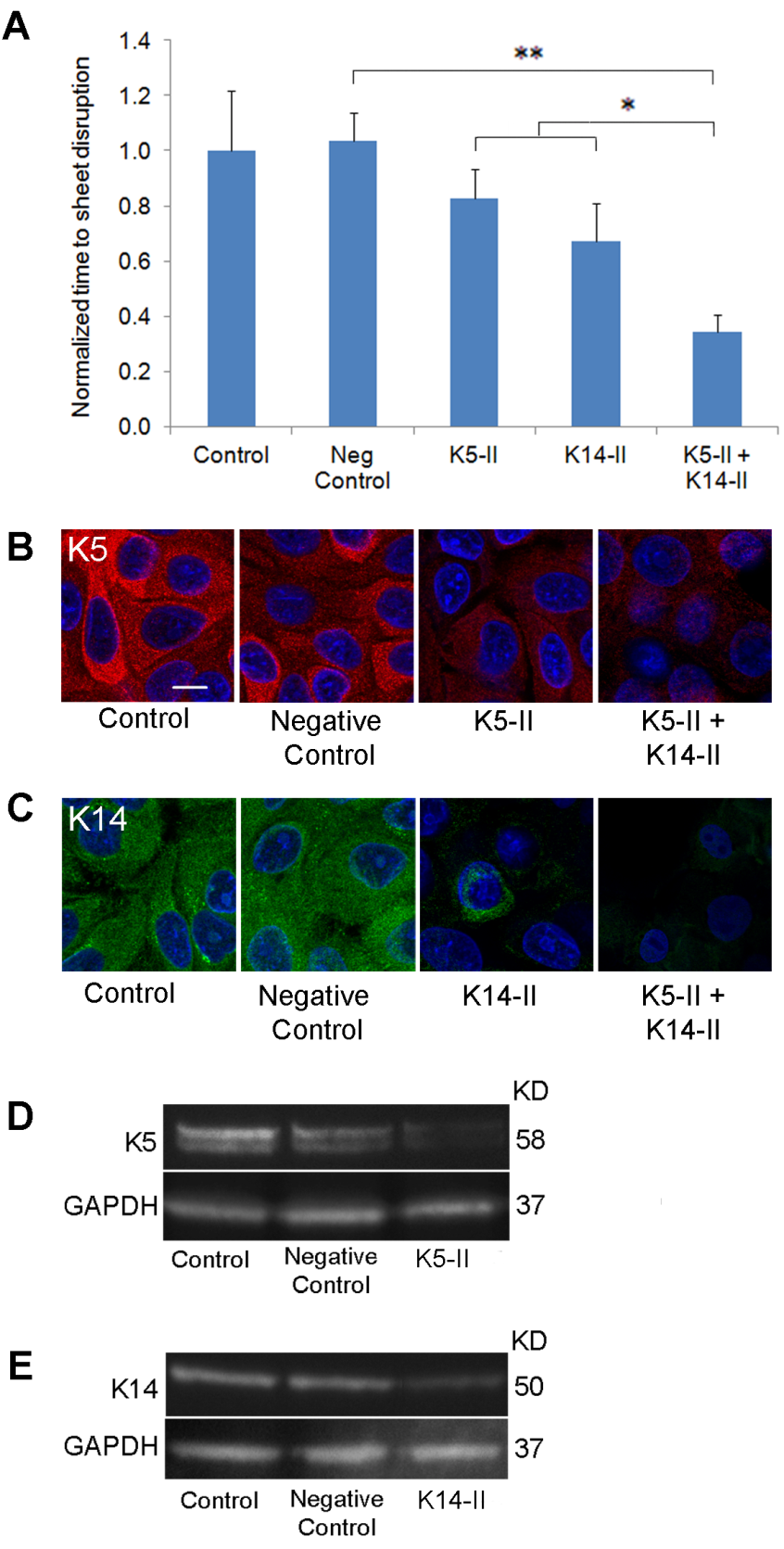


Supplementary Figure 1

Supplement: Additional file 1: Figure S1 — K5 and K14 siRNA expression and cohesion assay results using the second siRNA. (A) Shear test measuring cell sheet cohesion shows significant reduction in cohesion strength with double keratin knockdown. *p<0.05 and **p<0.01. Unnormalized times in seconds: control=145; negative control=150 (same controls as for Figure 2A); K5-II=120; K14-II=96; K5-II + K14-II=50. Immunostaining images of (B) K5 knockdown and (C) K14 knockdown. Scale bar is 10 μm. Western Blot analysis of (D) K5 knockdown and (E) K14 knockdown on adhered cells demonstrates reduction of K5 or K14 expression levels. GAPDH was used as a loading control. [file 1472-6750-13-17-S1.docx]

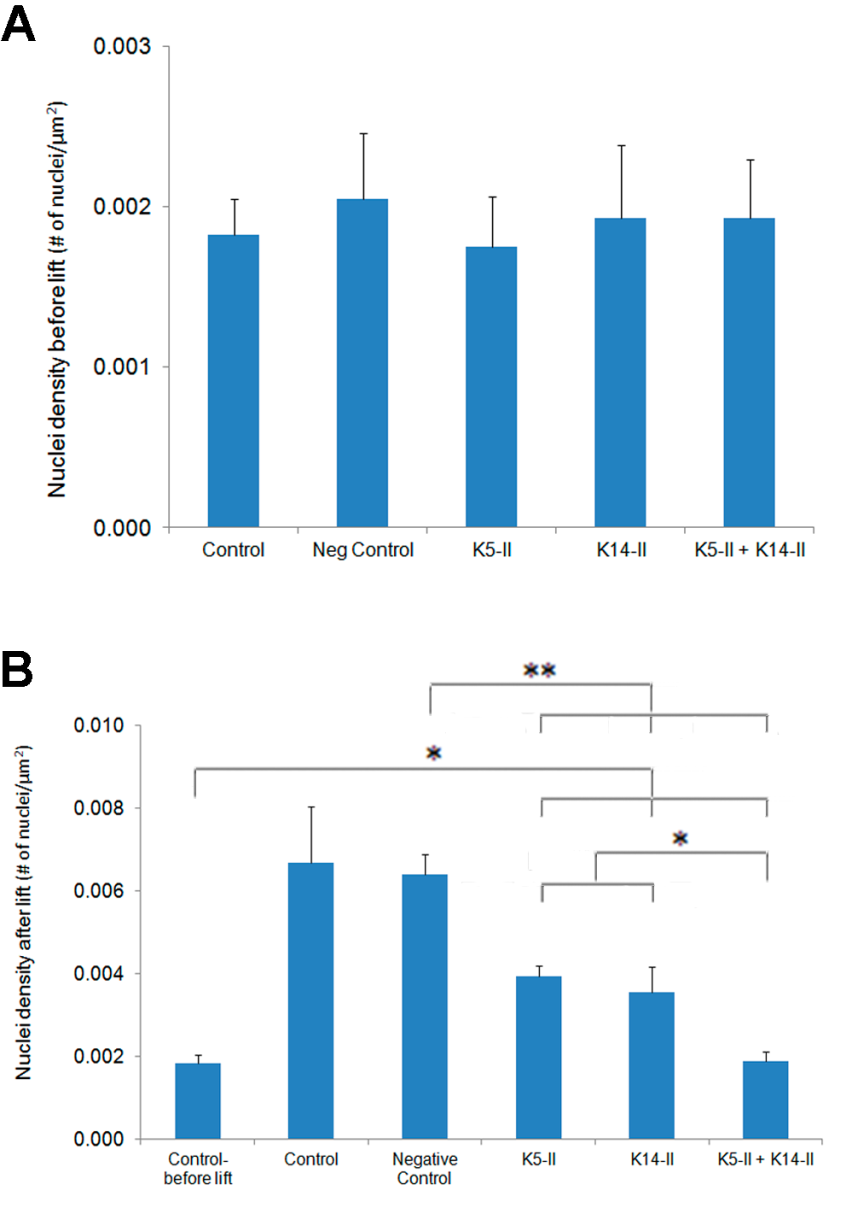


Supplementary Figure 2

Supplement: Additional file 2: Figure S2 — K5 and K14 siRNA contraction assay results using the second siRNA. (A) After siRNA transfection to suppress K5, K14, or both, without dispase lifting, no significant difference in nuclear density was observed among all treated samples. (B) After dispase lifting the siRNA transfected samples, all sheets contracted, but K5 and K14 siRNA treated contracted significantly less than the sample treated with negative control siRNA. *p<0.05, **p<0.01. [file 1472-6750-13-17-S2.docx]
